# Association between sensitivity to thyroid hormone indices and the risk of osteoarthritis: an NHANES study

**DOI:** 10.1186/s40001-022-00749-1

**Published:** 2022-07-11

**Authors:** Shuai Chen, Xiaohe Sun, Guowei Zhou, Jie Jin, Zhiwei Li

**Affiliations:** 1 Department of Orthopaedics, The Second Affiliated Hospital of Nanjing University of Chinese Medicine, Nanjing, China; 2 Department of Oncology, Jiangsu Province Hospital of Chinese Medicine, Affiliated Hospital of Nanjing University of Chinese Medicine, Nanjing, China; 3 Department of General Surgery, Jiangsu Province Hospital of Chinese Medicine, Affiliated Hospital of Nanjing University of Chinese Medicine, Nanjing, China

**Keywords:** Osteoarthritis, Thyroid, Sensitivity to thyroid hormone, NHANES

## Abstract

**Objectives:**

Thyroid hormones play an instrumental role in chondrogenic differentiation and matrix maturation. However, studies investigating the relationship between thyroid function and the risk of osteoarthritis (OA) remain scarce. This study was designed to investigate the correlation between thyroid status and OA from a novel perspective of sensitivity to thyroid hormones.

**Methods:**

The study included 8478 people from the National Health and Nutrition Examination Survey (NHANES) 2007–2010. The sensitivity to thyroid hormone indices included Thyrotroph Thyroxine Resistance Index (TT4RI), Thyroid-stimulating hormone (TSHI), Thyroid Feedback Quantile-based Index (TFQI), and Free Triiodothyronine /Free thyroxine (FT3/FT4), which were calculated based on serum free triiodothyronine (FT3), free thyroxine (FT4), and thyroid stimulating hormone (TSH). Considering the complex survey design and sample weights, we employed multivariate linear regression models and stratified analysis to evaluate the correlation between sensitivity to thyroid hormone indices and OA.

**Results:**

Study results indicated that participants with OA had elevated TT4RI, TSHI, and TFQI levels, and lower FT3/FT4 levels compared to those with non-arthritis. After adjusting for other covariates, FT3/FT4 was negatively associated with the risk of OA (OR = 1.162, 95%CI 1.048–1.478, *P* = 0.021); (OR = 1.261, 95%CI 1.078–1.623, *P* = 0.042). In subgroup analyses stratified by gender and BMI, participants with OA had higher TFQI levels compared to those without OA in both genders. (OR = 1.491, 95%CI 1.070–2.077, *P* = 0.018); (OR = 2.548, 95%CI 1.929–3.365, *P* < 0.001). The higher TFQI levels were consistently associated with the increased prevalence of OA in the BMI (< 18.5 kg/m^2^) group after adjusting for different covariates, but not in other BMI groups. In, addition, TFQI performed better than FT3/FT4, TSHI, and TT4RI on ROC analyses for OA prediction.

**Conclusions:**

The levels of FT3/FT4, TSHI, TT4RI, and TFQI are strongly associated with the prevalence of OA, which illustrates the complex correlation between the thyroid system and chondrogenic differentiation. TFQI may be used as a helpful indicator to predict OA and provide novel ideas for the evaluation and treatment of OA.

## Introduction

Osteoarthritis (OA) is a common chronic joint disease characterized by articular cartilage degradations and alterations in subchondral bone and synovium [1–3]. According to an epidemiological study published in 2020, approximately 3.8% of the global population (250 million people) are affected by OA, and 18% of females and 9.6% of males over 60 years old suffer from symptomatic OA [4, 5]. In addition to the huge physical burden on patients, OA is also a leading cause of rising medical costs in the United States [6]. Thus, early identification and intervention of OA are essential to reduce its prevalence.

Although the etiology and pathogenesis of OA have not yet been fully clarified, aging, obesity, infection, trauma, and metabolic disorders are considered risk factors for OA [7, 8]. Recent studies have demonstrated the important role of thyroid hormone metabolism in articular cartilage maintenance and the pathogenesis of OA [9–11]. Thyroid hormone is essential for endochondral ossification and the expression of genes that control chondrocyte maturation and matrix synthesis [12]. The lack of thyroid hormone results in the decrease of calcitonin secretion in the body, which affects calcium balance and bone metabolism, and eventually leads to the occurrence of OA [13]. Furthermore, clinical studies have shown that TSH levels are significantly positively correlated with the prevalence and progression of OA in patients with autoimmune thyroid disease (AITD) [14].

Thyroid function is evaluated clinically by measuring serum FT3, FT4, and TSH levels. However, FT3 or TSH alone may not be sufficient to reflect the regulation of thyroid hormone homeostasis [15]. Therefore, we proposed sensitivity to thyroid hormone indices such as TT4RI, TSHI, TFQI, and FT3/FT4 to comprehensively explain thyroid status [16, 17]. TT4RI, TSHI, and TFQI are introduced as quantitative markers for pituitary thyrotropic function [18, 19]. FT3 to FT4 ratio (FT3/FT4) can estimate the conversion efficiency of FT4 to FT3, which indirectly reflects the peripheral sensitivity of thyroid hormones. Increasing attention has been paid to the relationship between thyroid hormone sensitivity and metabolic disorders in recent years, and sensitivity to thyroid hormone indices have been proved to be reliable predictors of insulin resistance, type 2 diabetes (T2D), cardiometabolic risk, and disorders of glucose and lipid metabolism [20–22]. However, studies investigating the relationship between sensitivity to thyroid hormone indices and OA have not been reported. Therefore, we analyzed NHANES (2007–2010) data to explore the correlation between central and peripheral sensitivity to thyroid hormone and OA.

## Materials and methods

### Study population

The NAHNES is a research project of the National Center for Health Statistics (NCHS) that collects the health and nutrition statistics of the United States population. For the purpose of ensuring that the study participants are representative, the organization implemented a stratified, multistage, and clustered probability sampling design. In addition, all participants have written informed consent for data collection before the start of the investigation.

**Fig. 1:**
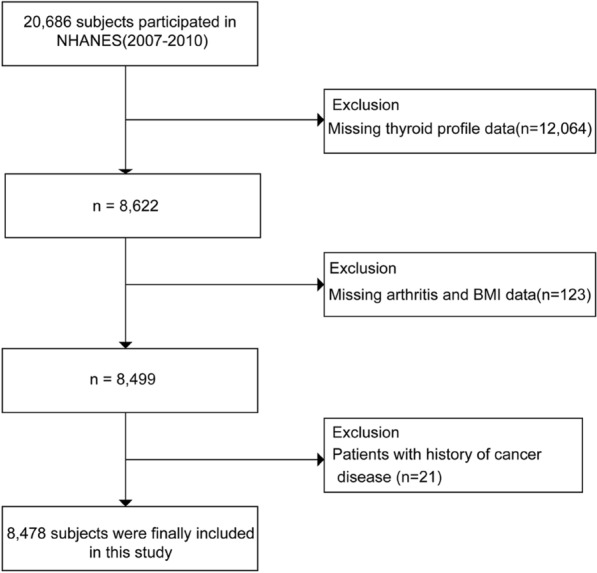
Study flowchart

In this research, we used demographic information, laboratory data, examination data, and questionnaire data from NHANES 2007–2010. We included participants who had completed nutrition investigations, medical examinations, and medical conditions questionnaires (*n* = 20,686). The exclusion criteria were as follows: (1) missing thyroid profile data (*n* = 12,064); (2) missing arthritis and BMI data (*n* = 123); (3) patients with a history of cancer diseases (*n *= 24). Thus, 8478 subjects were finally included in this analysis (Fig. 1).

### Definitions of osteoarthritis

Arthritis diagnosis data is part of self-reported personal interview data. Participants were asked if they had ever been told by their doctor or another health professional that they had arthritis. If “yes”, they were asked to classify their arthritis diagnosis as osteoarthritis (OA), RA (rheumatoid arthritis), psoriatic arthritis, and others.

### Assessment of thyroid function

The TT3, TT4, FT3 assay was a competitive binding immunoenzymatic assay. The TSH (mIU/L) level was detected by a two-site immunoenzymatic (“sandwich”) assay. The Tg (ng/mL) levels were assessed by a simultaneous one-step “sandwich” assay, while TgAb (IU/mL) and TPOAb (IU/mL) were assessed by a sequential two-step immunoenzymatic “sandwich” assay.

**The following is the calculation method of sensitivity to thyroid hormone indices:**

$$ {\text{FT3}}/{\text{FT4 ratio}}\, = \,{\text{FT3}}/{\text{FT4}} $$

A high FT3/FT4 ratio indicates a higher peripheral sensitivity to thyroid hormone [17, 23].

$$ {\text{TSHI}}\, = \,{\text{ln TSH}}\, + \,0.{1345 }*{\text{ FT4}} $$

$$ {\text{TT4RI}}\, = \,{\text{FT4 }}*{\text{ TSH}} $$

The higher TSHI and TT4RI indices indicate that the central sensitivity to thyroid hormone is lower.

$$ {\text{TFQI}}\, = \,{\text{cdfFT4}} - ({1} - {\text{cdfTSH}}) $$

TFQI was calculated with FT4 and TSH values according to the empirical cumulative distribution function. The advantage of TFQI is that it will not produce extreme values in the case of thyroid dysfunction and is more stable than TT4RI and TSHI. The TFQI value ranges from − 1 to 1. Depending on the previous studies, TFQI was negative, suggesting that the HPT axis is more sensitive to changes in FT4. A positive value indicated that the HPT axis is insensitive to FT4. 0 indicated that the sensitivity of the HPT axis to FT4 changes is normal.

### Covariates

The covariates are demographic information, laboratory data, and questionnaire data. Demographic information included age, gender, race, and education level. Laboratory data included HbA1c, albumin (g/dL), blood urea nitrogen (BUN, mg/dL), total calcium (mg/dL), creatinine (mg/dL), phosphorus (P, mg/dL), triglycerides (TG, mg/dL), and uric acid (UA, mg/dL). Finally, questionnaire data covered alcohol consumption, smoking behavior, physical activity, BMI, hypertension, and diabetes. Smoking behavior was defined as Current (smoked more than 100 cigarettes in the lifetime and currently still smoked), Past (smoked more than 100 cigarettes in the lifetime, but did not currently smoke), and Never (smoked less than 100 cigarettes in the lifetime). Alcohol consumption was classified as Non-drinker, low-to-moderate drinker (< 1 drink/day in women and < 2 drinks/day in men), and Heavy drinker (≥ 1 drink/day in women and ≥ 2 drinks/day in men). Physical activity was defined as Low (≤ twice a week), Moderate (< once a day and > twice a week), High (≥ once a day), and not recorded.

### Statistical analyses

The complex survey design factors involved in NHANES, including weights, clustering, and stratification, were all considered as recommended according to the analytical guideline regulated by NCHS. All analysis data conforming to normal or skewed distributions were presented as mean ± standard deviation, while categorical variables were shown as the number and percentage of subjects. The weighted chi-squared test and a weighted linear regression model were used to compare various indexes of the population between the “Arthritis and Non-arthritis” groups. The statistically significant covariates were then incorporated into the multiple linear regression model. By adjusting covariates, we further analyzed the correlation between sensitivity to thyroid hormone indices and OA, and calculated the effect value *β* and its 95% confidence intervals (CI). Furthermore, we performed subgroup analyses stratified by gender and BMI to enhance the confidence of the data. To evaluate the performance of the indices, we examined the receiver operating characteristics curves (ROC), which plot sensitivity against 1-specificity, and calculated the cut-points from ROC results. In this study, R software and EmpowerStats were used for data analysis, and *P* < 0.05 was considered statistically significant. Missing values often occur during the data extraction and data collection processes. For avoiding potential survival bias, we used multiple interpolations to replace the missing values in our study.

## Results

### Baseline characteristics of the participants with or without arthritis

We included 8478 participants in our study. The demographic characteristics of the research population were presented in Table 1. Compared to the non-arthritis group, participants in the arthritis group were more likely to be older, female, more educated, and non-Hispanic white. Meanwhile, significant differences were also observed between the two groups for smoking behavior, alcohol consumption, physical activity, BMI, WC, albumin, blood urea nitrogen, total calcium, creatinine, phosphorus, triglycerides, and serum uric acid (*P* < 0.001). In addition, participants in this study were divided into four categories: OA, rheumatoid arthritis, psoriatic arthritis, and other types according to the type of arthritis. In arthritic patients, the levels of FT4, Tg, TSHI, TT4RI, and TFQI were significantly higher, while the levels of FT3 and FT3/FT4 were significantly lower (*P* < 0.001).

**Table 1:** Baseline characteristics of the research population with and without arthritis

	Arthritis (*n* = 1282)	Non-arthritis (*n* = 7196)	*P* value
Age (years)	64.52 ± 12.78	40.95 ± 17.88	< 0.001
Gender			
Male	488 (38.07%)	3569 (49.60%)	< 0.001
Female	794 (61.94%)	3627 (50.41%)	
Race (%)			
Mexican American	57 (4.40%)	668 (9.27%)	< 0.001
Other race	94 (7.34%)	867 (12.05%)	
Non-Hispanic white	1012 (78.96%)	4882 (67.84%)	
Non-Hispanic black	119 (9.30%)	779 (10.83%)	
Level of education (%)			
Less than high school	395 (30.80%)	1163 (16.16%)	< 0.001
High school	370 (28.83%)	1493 (20.75%)	
More than high school	517 (40.37%)	4540 (63.09%)	
Smoking behavior (%)			
Current	733 (57.21%)	2840 (39.46%)	< 0.001
Past	290 (22.59%)	1906 (26.50%)	
Never	259 (20.20%)	2450 (34.04%)	
Alcohol consumption (%)			
Non-drinker	782 (60.98%)	4406 (61.23%)	< 0.001
Low to moderate drinker	407 (31.75%)	1328 (18.45%)	
Heavy drinker	93 (7.27%)	1462 (20.32%)	
Physical activity (%)			
Low	198 (15.41%)	1348 (18.73%)	< 0.001
Moderate	256 (19.95%)	1093 (15.20%)	
High	86 (6.74%)	400 (5.56%)	
Not recorded	742 (57.91%)	4355 (60.52%)	
BMI (kg/m^2^)	30.59 ± 7.28	27.69 ± 6.55	< 0.001
WC (cm)	104.42 ± 15.97	94.99 ± 16.54	< 0.001
HbAIC (%)	5.93 ± 0.89	5.51 ± 0.83	< 0.001
Albumin (g/dL)	4.14 ± 0.31	4.30 ± 0.33	< 0.001
Blood urea nitrogen (mg/dL)	15.61 ± 7.05	12.39 ± 4.84	< 0.001
Total calcium (mg/dL)	9.40 ± 0.42	9.45 ± 0.37	0.003
Creatinine (mg/dL)	0.94 ± 0.42	0.85 ± 0.27	< 0.001
P (mg/dL)	3.77 ± 0.58	3.85 ± 0.62	0.002
TG (mg/dL)	185.75 ± 185.77	150.17 ± 124.48	< 0.001
Uric acid (mg/dL)	5.73 ± 1.50	5.40 ± 1.37	< 0.001
FT3 (pmol/L)	4.65 ± 0.64	5.012 ± 0.84	< 0.001
FT4 (pmol/L)	10.62 ± 2.60	10.11 ± 2.02	< 0.001
TSH (mIU/L)	2.16 ± 2.59	2.06 ± 3.86	0.513
Tg (ng/mL)	19.60 ± 96.95	15.16 ± 31.60	0.007
TgAb (IU/mL)	16.11 ± 111.26	10.99 ± 97.56	0.202
TPOAb (IU/mL)	15.40 ± 77.07	20.01 ± 86.34	0.188
FT3/FT4	0.46 ± 0.11	0.51 ± 0.14	< 0.001
TSHI	1.90 ± 0.78	1.79 ± 0.71	< 0.001
TT4RI	21.73 ± 19.28	19.51 ± 19.14	0.004
TFQI	0.07 ± 0.35	− 0.01 ± 0.32	< 0.001
Type of arthritis (%)			
Osteoarthritis	630 (49.14%)	–	< 0.001
Rheumatoid arthritis	383 (29.88%)	–	
Psoriatic arthritis	8 (0.62%)	–	
Other	261 (20.36%)	–	

Mean ± SD for continuous variables: the *P* value was calculated by the weighted linear regression model. (%) for categorical variables: the *P* value was calculated by the weighted chi-square test

*BMI* body mass index, *WC* waist circumference, *P* phosphorus, *TG* triglycerides

### Associations between sensitivity to thyroid hormone indices and osteoarthritis

The adjusted correlation between OA and sensitivity to thyroid hormone indices was presented in Table 2. In the unadjusted model, we found that the TSHI, TT4RI, and TFQI were positively associated with the risks of OA, and FT3/FT4 was negatively associated with the prevalence of OA (*P* < 0.001). After adjusting for multiple covariates, FT3/FT4, TSHI, and TT4RI were not significantly associated with OA. However, the positive correlation between TFQI and the incidence of OA remained significant in the model 2 and 3. In addition, every 1 increase in TFQI was associated with a 26.1% increase in the risk of OA in the multivariable model 3 (OR = 1.261, 95% CI 1.079–1.623, *P* = 0.042). After converting TFQI to a categorical variable (quartiles), the TFQI levels of the Q2, Q4 groups were still positively correlated with the prevalence of OA in the multivariable model 3 compared with the lowest quartile of TFQI (Q1). In addition, the trend remained significant among different TFQI quartile groups (*P* < 0.05). The subgroup analyses stratified by gender and BMI were reported in Table 3. In both genders, those with higher TFQI levels had a higher incidence of OA than those with lower levels (OR = 1.491, 95% CI 1.070–2.077, *P* = 0.018) (OR = 2.548, 95% CI 1.929–3.365, *P* < 0.001). Furthermore, we discovered that higher TFQI levels were consistently associated with the increased risk of OA in the BMI (< 18.5 kg/m^2^) group after adjusting for different covariates, but not in other BMI groups.

**Table 2:** Associations between sensitivity to thyroid hormone indices and osteoarthritis

	FT3/FT4	TSHI	TT4RI	TFQI
Model 1, OR (95% CI, *P*)	0.007 (0.004, 0.016) < 0.001	1.288 (1.165, 1.424) < 0.001	1.007 (1.004, 1.010) < 0.001	2.028 (1.639, 2.509) < 0.001
Model 2, OR (95% CI, *P*)	1.062 (0.680, 1.658) 0.792	0.931 (0.842, 1.030) 0.167	0.999 (0.995, 1.003) 0.599	1.162 (1.048, 1.478) 0.021
Model 3, OR (95% CI, *P*)	1.063 (0.653, 1.730) 0.806	0.915 .018) 0.103	0.999 (0.995, 1.003) 0.484	1.261 (1.079, 1.623) 0.042

Logistic regression models:

Model 1: no covariates were adjusted

Model 2 was adjusted for demographic factors, including gender, age, and race

Model 3 was adjusted for gender, age, race, education, physical activity, smoking behavior, alcohol consumption, BMI, WC, HbA1c, albumin, blood urea nitrogen, total calcium, creatinine, phosphorus, triglycerides, and uric acid

**Table 3:** Subgroup analyses stratified by gender and BMI

	Model 1, OR (95% CI, *P*)	Model 2, OR (95% CI, *P*)	Model 3, OR (95% CI, *P*)
TFQI	2.028 (1.639, 2.509) < 0.001	1.162 (1.048, 1.478) 0.021	1.261 (1.079, 1.623) 0.042
TFQI (quartile)			
Q1	Reference	Reference	Reference
Q2	1.043 (0.841, 1.295) 0.700	1.243 (0.981, 1.576) 0.071	1.295 (1.011, 1.658) 0.041
Q3	1.303 (1.059, 1.602) 0.012	1.298 (1.029, 1.638) 0.028	1.360 (1.068, 1.731) 0.013
Q4	1.622 (1.328, 1.980) < 0.001	1.283 (1.020, 1.612) 0.033	1.364 (1.073, 1.736) 0.011
* P* for trend	< 0.001	0.05	0.019
Subgroup analysis stratified by gender			
Male	1.491 (1.070, 2.077) 0.018	1.383 (0.961, 1.989) 0.081	1.435 (0.980, 2.102) 0.064
Female	2.548 (1.929, 3.365) < 0.001	1.013 (0.736, 1.396) 0.935	1.201 (0.851, 1.695) 0.298
Subgroup analysis stratified by BMI			
BMI < 18.5	1.961 (1.581, 2.433) < 0.001	1.173 (1.023, 1.493) 0.019	1.224 (1.052, 1.574) 0.012
BMI 18.5–23.9	1.765 (1.048, 2.972) 0.032	1.740 (0.974, 3.109) 0.061	1.373 (0.745, 2.531) 0.309
BMI 24–28	2.672 (1.704, 4.191) < 0.001	1.101 (0.660, 1.836) 0.713	1.096 (0.638, 1.883) 0.741
BMI > 28	1.875 (1.416, 2.481) < 0.001	1.050 (0.768, 1.436)0.758	1.219 (0.875, 1.698) 0.242

Logistic regression models:

Model 1: no covariates were adjusted

Model 2 was adjusted for demographic factors, including gender, age, and race

Model 3 was adjusted for gender, age, race, education, physical activity, smoking behavior, alcohol consumption, BMI, WC, HbA1c, albumin, blood urea nitrogen, total calcium, creatinine, phosphorus, triglycerides, and uric acid

### ROC curves for optimal cut-points of sensitivity to thyroid hormone indices

**Fig. 2:**
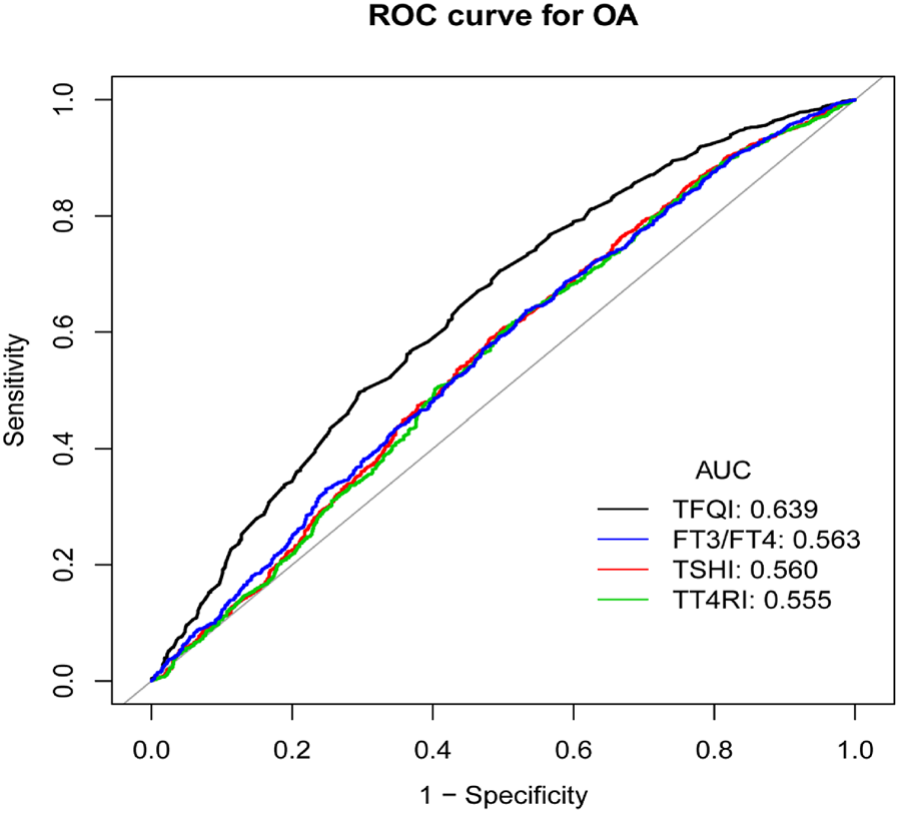
ROC curves for optimal cut-points of sensitivity to thyroid hormone indices

Figure 2 shows that the areas under the curve (AUC) for FT3/FT4, TSHI, TT4RI, and TFQI were 0.563 (95% CI 0.542–0.584), 0.560 (95% CI 0.539–0.581), 0.555 (95% CI 0.533–0.576), 0639 (95% CI 0.619–0.659). TFQI performed better than FT3/FT4, TSHI, TT4RI on ROC analyses for OA prediction. The optimal cut-points of TFQI for OA prediction was 0.450, and sensitivity and specificity of this cutoff point were 0.705 and 0.505, respectively.

## Discussion

In this study, we investigated the relationship between sensitivity to thyroid hormone and the prevalence of OA based on the NHANES 2007–2010 data. We demonstrated that both peripheral and central thyroid resistance indices were strongly correlated with the prevalence of OA. The higher levels of FT3/FT4 and lower levels of TSHI, TT4RI, and TFQI were associated with increased OA prevalence. In subgroup analyses stratified by BMI, the strongest relationship between TFQI and OA prevalence was observed in the BMI (< 18.5 kg/m^2^) group. The innovation of this study is that we used sensitivity to thyroid hormone indices rather than FT3, FT4, and TSH as predictors of bone loss for the first time. We directly linked resistance to thyroid hormone with chondrogenic differentiation, which provides a novel hypothesis for the early screening and treatment of OA.

We observed that higher FT3 and FT4 levels were associated with an increased risk of OA in this study. T4 is generally considered the primary hormone produced and released by the thyroid gland, and most T4 is thought to be converted to T3 by a cytoplasmic deiodination enzyme (D2) before it enters cell nuclei to bind to nuclear receptors and alter gene expression [24]. T3 could stimulate the synthesis of collagen X and the expression of matrix metalloproteinase 13 (MMP-13) and alkaline phosphatase, and promoted the progress of MMP-13, ultimately leading to cartilage mineralization and degradation [25–27]. Kim et al. indicated that both hypothyroid and hyperthyroid states were associated with the incidence of knee arthralgia and knee osteoarthritis [10]. According to Devrimsel et al., patients with hypothyroidism had thinner femurs and a higher risk of OA than healthy individuals at all measurement sites [28]. However, a prospective cohort study showed no association between hypothyroidism or hyperthyroidism measured by TSH and the risk of total hip replacement (THR) or total knee replacement (TKR) due to OA [29, 30]. In addition, several meta-analyses have shown that the presence of AITD not only increases the risk of clinically significant thyroid disease, but may also be associated with an increased prevalence of OA, RA, and other musculoskeletal diseases [31, 32]. Contrary to most previous studies, our result showed no relationship between TgAb, TPOAb, and the risk of OA. We considered that the discrepant results of these studies may be related to the different criteria for determining thyroid function and the composition of participants. In addition, TSH, FT4, and FT3 are highly interactive with each other, and there are complex interactions in the HPT axis [32]. Therefore, the measurement of individual parameters might be insufficient to explain the relationship between the thyroid system and chondrocyte differentiation. We began to use composite indices for further evaluation.

Our study observed that levels of sensitivity to thyroid hormone indices (central and peripheral) were associated with the prevalence of OA. The study by Nie et al. demonstrated a positive correlation between FT3/FT4 levels and serum osteocalcin levels, suggesting FT3/FT4 may affect serum osteocalcin levels in healthy thyroid subjects [33, 34]. Osteocalcin has been proposed as a biomarker for OA detection and monitoring. Its biologic function is considered to be related to the inhibition of bone matrix mineralization [35, 36]. In addition, multiple cross-sectional studies have found that FT3/FT4, TSHI, and TT4RI are significantly correlated with chronic kidney disease (CKD), non-alcoholic fatty liver disease (NAFLD), and coronary artery disease (CAD) [37–39]. As described in this article, we found that FT3/FT4 levels decreased with increasing risk of OA, whereas TSHI and TT4RI levels increased with increasing OA prevalence in the subjects. Taken all into consideration, these evidences showed the potential role of sensitivity to thyroid hormones in the prediction and evaluation of OA.

A previous study suggested that TFQI is more stable than TSH in evaluating sensitivity to thyroid hormone [20]. The study by Sun et al. demonstrated a close relationship between TFQI and obesity, metabolic syndrome, and diabetes-related mortality [40]. Consistent with these studies, our result showed that elevated TFQI was associated with an increased risk of OA and this positive association was still significant after adjusting for multiple covariates. Therefore, we hypothesized that chondrocyte genesis and differentiation may be related to the central sensitivity to thyroid hormone. In the subgroup analysis stratified by BMI, we found that the higher TFQI levels were consistently associated with a decreased risk of OA in the BMI (< 18.5 kg/m^2^) group, but not in other BMI groups. This suggested that thyroid hormone resistance may be affected by fat accumulation. A previous study by Zhou et al. found that obese patients had higher TSHI and TFQI levels compared to the control group, and TSHI and TFQI decreased significantly over time after bariatric surgery [41]. In addition, a cross-sectional analysis also showed that the secretory capacity of the thyroid gland (SPINA-GT) and TSHI were significantly negatively related to obesity indices, suggesting that obesity reduces levels of thyroid homeostasis indices [42].

Our study has some strengths. In our research, we used nationally representative NHANES data and combined sample weights for statistical analysis to ensure the authenticity and objectivity of the data. We employed innovative thyroid hormone indices and achieved significant results consistent with previous studies, but there were also some limitations. First, the anti-osteoarthritis treatment history of our participants was absent, which affects the objectivity of the results. In addition, the diagnosis of arthritis was based on patients’ self-report which may lead to bias. Finally, we used a cross-sectional approach in this study, thus we could not collect patient follow-up data to explore the causal relationship between STTH and OA.

## Conclusions

Among our representative US population, our present study concluded that TFQI was negatively associated with the risk of OA after multiple adjustments. Therefore, we hypothesized that TFQI could be used as a new predictor of OA, providing help for early screening and preventive of OA.

## Data Availability

The survey data are publicly available on the internet for data users and researchers throughout the world http://www.cdc.gov/nchs/nhanes/.
